# Transcription Initiation by Mix and Match Elements: Flexibility for Polymerase Binding to Bacterial Promoters

**Published:** 2007-12-17

**Authors:** India G. Hook-Barnard, Deborah M. Hinton

**Affiliations:** Gene Expression and Regulation Section, Laboratory of Molecular and Cellular Biology, National Institute of Diabetes Digestive and Kidney Diseases, National Institutes of Health, Bldg. 8 Room 2A-13, Bethesda, MD 20892-0830

**Keywords:** polymerase, sigma70, promoter, transcription

## Abstract

Bacterial RNA polymerase is composed of a core of subunits (β, β′, α_1_, α_2_, ω), which have RNA synthesizing activity, and a specificity factor (σ), which identifies the start of transcription by recognizing and binding to sequence elements within promoter DNA. Four core promoter consensus sequences, the −10 element, the extended −10 (TGn) element, the −35 element, and the UP elements, have been known for many years; the importance of a nontemplate G at position −5 has been recognized more recently. However, the functions of these elements are not the same. The AT-rich UP elements, the −35 elements (^−35^TTGACA^−30^), and the extended −10 (^−15^TGn^−13^) are recognized as double-stranded binding elements, whereas the −5 nontemplate G is recognized in the context of single-stranded DNA at the transcription bubble. Furthermore, the −10 element (^−12^TATAAT^−7^) is recognized as both double-stranded DNA for the T:A bp at position −12 and as nontemplate, single-stranded DNA from positions −11 to −7. The single-stranded sequences at positions −11 to −7 as well as the −5 contribute to later steps in transcription initiation that involve isomerization of polymerase and separation of the promoter DNA around the transcription start site. Recent work has demonstrated that the double-stranded elements may be used in various combinations to yield an effective promoter. Thus, while some minimal number of contacts is required for promoter function, polymerase allows the elements to be mixed and matched. Interestingly, which particular elements are used does not appear to fundamentally alter the transcription bubble generated in the stable complex. In this review, we discuss the multiple steps involved in forming a transcriptionally competent polymerase/promoter complex, and we examine what is known about polymerase recognition of core promoter elements. We suggest that considering promoter elements according to their involvement in early (polymerase binding) or later (polymerase isomerization) steps in transcription initiation rather than simply from their match to conventional promoter consensus sequences is a more instructive form of promoter classification.

## The Multi-Step Process of Transcription Initiation

**Figure 1:**
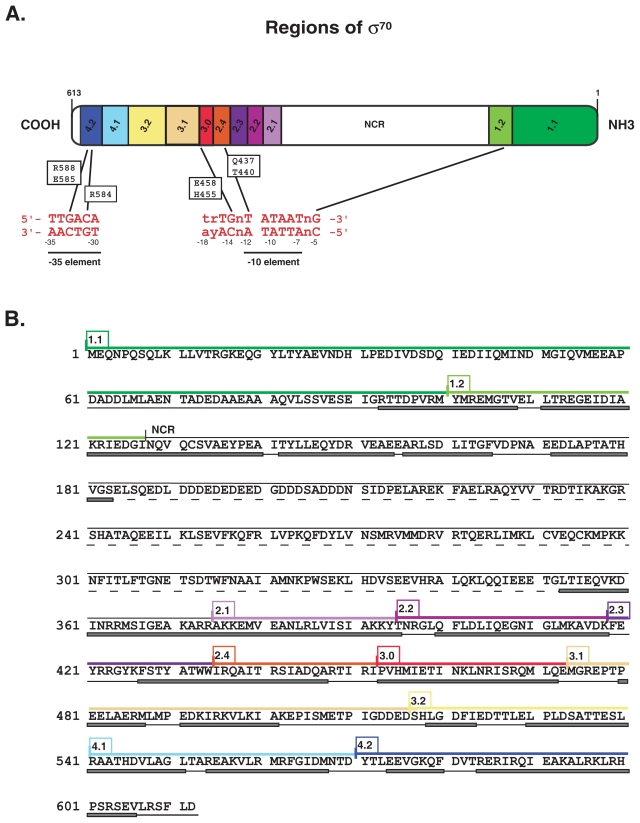
**(A)** Schematic of *E. coli* σ^70^. Subregions (1.1, 1.2, etc) and non-conserved region (NCR) are based on function, structure, and sequence conservation (Gruber and Gross, 2003). Canonical promoter sequence elements are shown below sigma. Specific σ^70^ residues, which are thought to interact with base determinants, are indicated (see text for details). **(B)** Amino acid sequence of *E. coli* σ^70^. Subregions of σ^70^ are indicated above the sequence and are colored as in A. Below the sequence, secondary structure is represented as gray boxes (a-helices), black lines (coils) and dashed lines (disordered structure). (See (Gruber and Gross, 2003; Vassylyev et al. 2002) and text for details.)

The process of transcription begins when RNA polymerase recognizes and binds to DNA elements within a promoter sequence (reviewed in (Browning and Busby, 2004; Murakami and Darst, 2003; Young et al. 2002)). In bacteria, RNA polymerase is composed of a core of multiple subunits (β, β′, α_1_, α_2_, and ω) that is tightly associated with a σ specificity factor. While core contains the active site for polymerase and thus is capable of synthesizing RNA, the sigma factor controls when and where transcription is initiated. Prokaryotes have multiple sigma factors; a primary σ that is needed for the expression of housekeeping genes during exponential growth and alternate σ factors that are used under certain conditions of growth or stress (Gruber and Gross, 2003; Paget and Helmann, 2003). The primary σ factor in *E. coli* is σ^70^. Like other primary σ factors, σ^70^ can be divided into four major domains (regions 1, 2, 3, and 4) and subdomains (2.1, 2.2, 2.3, etc), based on function, structure, and sequence conservation (Gruber and Gross, 2003; Lonetto et al. 1992) (Fig. 1A and B).

**Figure 2:**
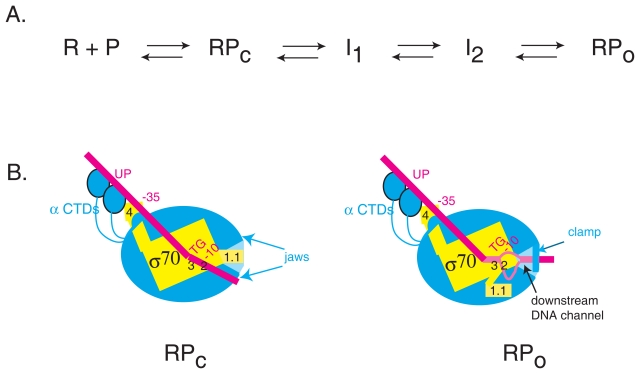
**(A)** Steps in transcription initiation. R (polymerase) and P (promoter) first interact to form a closed complex (RPc). This complex then proceeds through intermediates (I_1_ and I_2_) to form the open complex (RPo). **(B)** Cartoon depicting polymerase promoter contacts in RPc and RPo. Core polymerase (subunits β, β^1^, α_2_, and ω) is shown in teal, σ^70^ is shown in yellow, and the DNA is shown in magenta. Interactions between the C-terminal domains of the α subunits (α CTDs) and the UP element, σ^70^ region 4 and the −35 element, σ^70^ region 3 and the ^−^15TGn^−^13 element, and σ^70^ region 2 and the −10 element are indicated. RPc is a closed complex in which the DNA has not yet entered the primary channel. Full entry of DNA into the channel is blocked by the presence of σ^70^ region 1.1. In RPo, σ^70^ region 1.1 has moved, the DNA from around −11 to +3 is unwound, the template strand has descended into the active site of polymerase, and a portion of β′, called the clamp, has secured the downstream DNA (Kontur et al. 2006). See text for details and additional references.

Detailed studies of transcription initiation at a few well-characterized promoters have demonstrated that initiation by *E. coli* RNA polymerase is a multi-step process (Fig. 2) that requires multiple contacts between the polymerase and the DNA (Buc and McClure, 1985; Craig et al. 1998; Davis et al. 2007a; Kontur et al. 2006; Schickor et al. 1990; Sclavi et al. 2005; Spassky et al. 1985). Each step on the path from free RNA polymerase and promoter to the final transcriptionally competent complex is an opportunity to regulate the initiation process. In addition to biochemical and kinetic studies, structural analyses using polymerase, core, and the primary σ of thermophilic bacteria with and without DNA have provided a detailed model upon which to conceptualize these steps (Campbell et al. 2002; Murakami et al. 2002a; Murakami et al. 2002b; Murakami and Darst, 2003; Vassylyev et al. 2002; Young et al. 2002). The structure of a portion of *E. coli* σ^70^ has also been obtained (Malhotra et al. 1996). Thus, we can now generally chart the changes in DNA and polymerase that occur during initiation. In this review, we summarize data that addresses the contribution of individual promoter elements to the process of transcription initiation. However, to understand these interactions, we must first discuss the identified steps in transcription initiation that lead to the start of transcription.

### The closed (RPc) polymerase/promoter complex

Initial recognition and binding of promoter DNA (P) by RNA polymerase (R) is referred to as closed complex, RPc (Fig. 2), because the DNA is fully double-stranded (ds) or closed. In RPc the polymerase specifically recognizes ds binding elements and is able to form a complex with the DNA detectable by electrophoretic mobility shift assay (EMSA). As we detail in sections below, specific promoter elements that may be used for recognition include: UP elements (positioned between −60 and −40) that are bound by the C-terminal domains of the α subunits of core (α-CTDs), a −35 element that is recognized by residues in σ^70^ region 4, a TGn motif that is recognized by residues in σ^70^ region 3, and sequences within a −10 element, primarily the base pair at −12, that is recognized by residues in σ^70^ region 2 (Fig. 1 and 2). Although the RPc complex is specific, it is also readily reversible and therefore, easily challenged by the presence of competitor such as nonspecific DNA or the polyanion heparin. Footprinting data demonstrate that the polymerase initially protects the DNA from cleavage from at least −55 to +5. However, this short footprint is only seen for the earliest closed complex when footprints are carried out at lower temperatures (e.g. 8 °C) (Schickor et al. 1990) or with polymerase mutants (Cook and deHaseth, 2007) that trap the complex at this stage of the process. This protection pattern is consistent with the idea that in RPc the promoter DNA lies outside of the polymerase primary channel and is mostly undistorted (Kontur et al. 2006; Mekler et al. 2002; Murakami and Darst, 2003).

### Kinetic intermediates on the path from RPc to RPo

For transcription initiation to occur, RPc must transition into a species, RPo, in which the DNA is both bent and unwound (open), and polymerase has undergone major conformational changes, called isomerization. Kinetic analyses (Buc and McClure, 1985; Craig et al. 1998; Davis et al. 2007a; Kontur et al. 2006; McKane et al. 2001; Saecker et al. 2002; Schickor et al. 1990; Sclavi et al. 2005; Spassky et al. 1985; Straney and Crothers, 1985; Straney and Crothers, 1987a; Straney and Crothers, 1987b) have revealed two significant intermediates in the pathway from RPc to RPo. For the lambda promoter P_R_, the first intermediate I_1_ is characterized by a bending of the DNA and the extension of the footprint to +20. Recent work suggests that this early step may be facilitated by the presence of far upstream DNA via interaction with other components of core (Davis et al. 2005; Davis et al. 2007a; Ross and Gourse, 2005). Wrapping of upstream DNA has been proposed to reposition portions of the β and β′ subunits called the jaws, widening the primary channel, and allowing the DNA to move into the downstream end of this channel (Saecker et al. 2002) (Davis et al. 2007b). Alternatively, (or in addition) contacts between the far upstream DNA and the α-CTD of RNAP may aid in this transition (Ross and Gourse, 2005). However, despite these changes, in I_1_ the polymerase has not yet fully undergone its isomerization process, and, thus, I_1_ is competitor sensitive.

A later intermediate for lambda P_R_, I_2_, requires a large conformational change in polymerase. Because σ^70^ region 1.1 (the N-terminal 100 residues of σ^70^) is thought to reside within the primary channel in the absence of DNA (Kontur et al. 2006; Mekler et al. 2002; Murakami and Darst, 2003), region 1.1 must relocate to make the channel fully accessible for the DNA (Fig. 2B). The movement of region 1.1 out of the channel is proposed to be coupled to the late folding of portions of the β′ subunit, designated the ‘clamp’. Movement of region 1.1 can affect the rate of the transition from RPc to RPo, but this modulation varies with the promoter context (Hook-Barnard and Hinton, unpublished) (Vuthoori et al. 2001; Wilson and Dombroski, 1997). Also, in I_2_, DNA downstream of −12 begins to unwind and the template strand begins its decent in the active site (Kontur et al. 2006). Evidence indicates that the −11A in the −10 element is crucial for instigating the melting process, which then propagates downstream through the start site (Fenton and Gralla, 2001; Lim et al. 2001; Tsujikawa et al. 2002). It is currently unclear which begins first, movement of region 1.1 out of the channel or DNA melting. However, the movement of region 1.1, together with DNA melting appears to be the rate-limiting step, and the order may be determined by the promoter context.

### The open (RPo) polymerase/promoter complex

The stable RPo species is achieved when the DNA around the transcriptional start site is fully separated, creating a transcription bubble from around −11 to +3, the template strand is located in the active site with the +1 nucleotide ready for base paring with incoming NTPs, and the polymerase ‘clamp’ fully closes onto the DNA that is lying in the DNA channel (Fig. 2B) (Brodolin et al. 2005; Davis et al. 2007a; Kainz and Roberts, 1992; Lim et al. 2001; Nguyen and Burgess, 1997; Sasse-Dwight and Gralla, 1989; Sasse-Dwight and Gralla, 1991). In RPo, the nontemplate strand nucleotides of the −10 element interact with σ region 2.3, stabilizing the polymerase-promoter complex. Additionally, the nucleotide at −5 may be recognized by σ^70^ region 1.2 (Feklistov et al. 2006; Haugen et al. 2006; Zenkin et al. 2007). RPo is the primary species at 37 °C and the DNase footprint at RPo typically extends from −55 to +25 (Davis et al. 2007a; Schickor et al. 1990). RPo is usually resistant to competition, although the stability of this complex varies with the promoter context (Haugen et al. 2006; Vuthoori et al. 2001).

## Double-Stranded Promoter Elements Involved in Transcription Initiation

**Figure 3:**
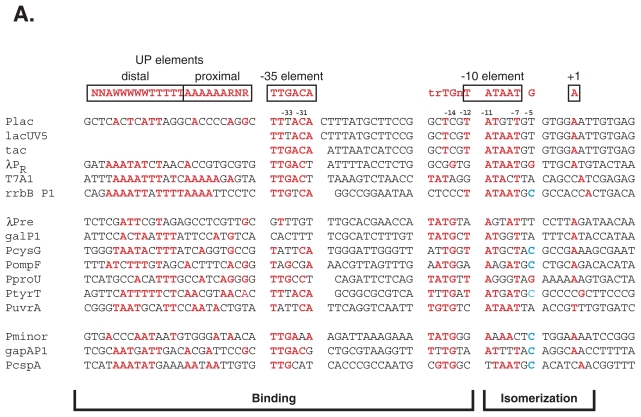
Sequences of various σ^70^-dependent promoters. Consensus sequences of DNA elements are shown at the top in red. (Sequences are given 5′ 3′ of the nontemplate (top) strand; W = A or T; R = A or G; Y= C or T; N = any nucleotide) *E. coli* promoters are from the *E. coli* genome (accession number NC000913): Plac, 365627-365559; rrnBP1, 4164330–4164396; galP1, 791363-791296; PcysG, 3495716-3495783; PompF, 986374-986307; PproU, 2802718-2802784; PtyrT, 1286947-1286881; PuvrA, 4272014-4271947; gapAP1, 1860700–1860767; PcspA, 3717854-3717919. The bp changes present in the Plac derivatives lacUV5 (Stefano et al. 1980) and Ptac (Mulligan et al. 1985) are indicated. λ promoters are from the λ genome (accession number J02459.1): P_R_, 37964-38031; Pre, 38402-38335. Pminor is from the T4 genome (accession number AF158101.6; positions 23736-23670). T7A1 is from the T7 genome (accession number AY264774.1; positions 438–505). (It should be noted that among various references, promoter sequences with the same name can differ at the farthest upstream and downstream portions of the sequences because of differences in the length of DNA cloned for the particular study.) Matches to consensus sequences within the various promoter sequences are given in red. For the −5 contact, the preferred base is a G (shown in red) and the highly nonpreferred base is a C (shown in blue). The involvement of portions of promoter DNA with earlier steps of transcription initiation (polymerase binding) or later steps (polymerase isomerization) is indicated at the bottom.

For more than 25 years, it has been known that σ^70^-dependent promoters require specific sequences for efficient transcription. Early work revealed consensus sequences at the −35 (^−35^TTGACA^−30^) and −10 (^−12^TATAAT^−7^) regions of σ^70^-dependent promoters as well as a weaker consensus of ^−17^trTGn^−13^ (Fig. 3) ((Harley and Reynolds, 1987; Hawley and McClure, 1983; Lisser and Margalit, 1993; Lisser and Margalit, 1994; Siebenlist and Gilbert, 1980; Siebenlist et al. 1980); reviewed in (Campbell et al. 2002; Gruber and Gross, 2003; Murakami et al. 2002a)). Subsequent genetic, biochemical, and structural studies have demonstrated that specific residues within σ^70^ interact both directly and indirectly with bases within these consensus elements (Fig. 1).

### Recognition of the −35 element

For the −35 element, specific substitutions within σ^70^ region 4.2 have been shown to relax the specificity of polymerase for bps at positions −33 (E585Q, R588H) (Gardella et al. 1989; Keener and Nomura, 1993) and −31 (R584C) (Gregory et al. 2005; Siegele et al. 1989) (Fig. 1A). In addition, *in vitro,* polymerase containing the σ^70^ R584C or R588H mutation shows the same relaxed specificity (Makela and Hinton, unpublished), and polymerase with an σ^70^ R584A substitution prefers a −31 A:T bp rather than the wild type C:G (Gregory et al. 2005). These results have suggested that σ^70^ residues R584, E585, R588 interact directly with base determinants at these positions. The structure of *Thermus aquaticus* σ region 4 in a complex with −35 DNA element DNA has revealed an extensive interaction between 11 residues in σ^70^ region 4 and 9 bp of the −35 element DNA, either via specific contact of base determinants, water mediated contact, or contact with the DNA backbone (Campbell et al. 2002). Consistent with the previous work, the specific contacts with template base determinants are seen at positions −31 and −33 with residues R584 and E585, respectively. These residues lie within the second helix of a classic DNA binding helix-turn-helix motif. The structure does not show a specific contact between R588 and a −35 bp element, but suggests that the position of R588 could affect E585, (Campbell et al. 2002) or perhaps it makes direct contact in the context of the entire polymerase.

### Recognition of the −10 element

Although the −10 element (^−12^TATAAT^−7^) is widely accepted as being crucial for promoter recognition, the −12 T: A position is the only base pair for which suppressor amino acids have been found. Both Q437H (Waldburger et al. 1990) and T440I (Siegele et al. 1989) within σ^70^ region 2.4 suppress a T:A to C:G mutation at position −12 (Fig. 1A). The 6.5 Å resolution structure of *T. aquaticus* polymerase with DNA (Murakami et al. 2002a) is consistent with this work. It shows residues that correspond to Q437 and T440 in *E. coli* σ^70^ positioned on the same side of an α helix facing the major groove at position −12. Another study has shown that mutations at σ^70^ residues W434, R436, R441, or R451 can impair binding of polymerase to a duplex promoter sequence from −41 to −12 (Fenton et al. 2000), suggesting that residues both in σ^70^ region 2.4 and in region 2.3 can influence binding to position −12. Recent work using polymerase with quadruple mutations in region 2.3 also argues that this region of σ^70^ may contact portions of the −10 element while it is in ds form (Cook and deHaseth, 2007).

Further information about σ^70^ recognition of ds DNA within the −10 element has been obtained using a portion of free σ^70^. Although full length σ^70^ does not bind DNA, a weak interaction of σ^70^ lacking region 1.1 with ds DNA can be detected using a nitrocellulose filter binding assay (Dombroski et al. 1992). With this assay, Dombroski (Dombroski, 1997) demonstrated that within the −10 element, only bp mutations at positions −12, −11, or −10 were deleterious for binding by a σ^70^ peptide containing regions 2 through 4, suggesting again that it is the upstream portion of this element that is specifically recognized as ds DNA.

### Recognition of the ^−15^TGn^−13^ element

A third consensus element recognized by σ^70^ is the ^−15^TGn^−13^ motif, also called the extended −10 sequence (Keilty and Rosenberg, 1987). Optimal activity requires the presence of both the −15 T:A and −14 G:C; although depending on the promoter, the presence of just one of these bp can be advantageous for promoter activity even when the other is mutated (Burr et al. 2000; Grana et al. 1985; Keilty and Rosenberg, 1987; Mitchell et al. 2003). Biochemistry and suppressor genetics, including comprehensive alanine mutagenesis throughout region 3 and *in vitro* transcription assays using mutant σ^70^ proteins, have indicated that residues E458 and H455 within σ^70^ region 3.0 interact with the −14G: C base pair (Barne et al. 1997; Bown et al. 1999; Sanderson et al. 2003) (Fig. 1A). Glutamic acid residues are known to interact with a cytosine determinant, such as the interaction of σ^70^ region 4 E585 with the C of the G:C bp at position −33, suggesting that E458 may contact the C base determinant of the G:C bp at position −14 (Barne et al. 1997). Furthermore, the *T. aquaticus* polymerase/promoter structure (Murakami et al. 2002a) shows the residues analogous to H455 and E458 lying along the surface of the region 3 α-helix and toward the major groove of the extended −10 element. Mutation analyses have also revealed that residues 455 and 458 in region 2.4 influence interaction of polymerase with the TGn motif. Thus, it has been suggested that the two α-helices, σ^70^ regions 2.4 and 3, comprise a pincer for the ds binding element of the TGn motif (Sanderson et al. 2003). As such, regions 2.4 and 3 together would constitute a recognition domain for a ds binding promoter element ^−15^TgnT^−12^ that includes both the TGn and the T at position −12.

In gram-positive bacterial promoters, the extended −10 motif extends to −17, with a consensus sequence of ^−17^TRTG^−14^ (Helmann, 1995; Moran et al. 1982; Voskuil and Chambliss, 2002). For *E. coli* polymerase, the specific sequence at −17 and −16 can affect the level of transcription (Burr et al. 2000; Mitchell et al. 2003), but there is no evidence that these positions are contacted directly.

### Recognition of the UP elements by the C-terminal domains of the α subunits

An early list of σ^70^-dependent promoter sequences revealed an A/T rich sequence upstream of the −35 element that was present enough to seem significant (Hawley and McClure, 1983). In addition, sequences within this region were found to significantly enhance transcription from some promoters (Chan and Busby, 1989) (also (Ross et al. 1993) and references therein). Using the ribosomal promoter rrnB P1, Ross and co-workers demonstrated that the region from −40 to −60, termed an UP element, was specifically bound by the C-terminal domain of the α-subunits of polymerase (Gaal et al. 1996; Ross et al. 1993) Subsequent work has shown that 2 subsites of A/T rich sequences, a proximal UP element (−45 to −37) and a distal UP element (−58 to −45) (Estrem et al. 1998) are contacted through minor groove interactions with the DNA (Naryshkin et al. 2000; Ross et al. 2001) (Fig. 3, numbering is relative to the −35 element of ^−35^TTGACA^−30^). Each element can be contacted by one of the two α subunits present in polymerase, and whether the proximal, distal or both elements are contacted can affect promoter recognition and activity (Cellai et al. 2007; Typas and Hengge, 2005). Although a specific sequence is preferred for binding, the α subunits will also interact nonspecifically with this region of DNA (Burns et al. 1999; Ross and Gourse, 2005).

## Interaction of Polymerase with Single-Stranded DNA Elements

### Interaction of σ^70^ region 2.4 with an unpaired base at position −12

Although the T:A bp at position −12 is recognized by σ^70^ region 2.4 within the context of ds DNA, several lines of evidence indicate that the nontemplate −12 T may also be recognized when it is ss. Marr and Roberts (Marr and Roberts, 1997) demonstrated that RNA polymerase specifically recognizes and binds to a ss oligomer containing the nontemplate sequence of the −10 element and that a −12 T to C mutation within this oligomer is suppressed by the same σ^70^ substitution, Q437H, that suppresses the −12 T:A to C:G bp change in ds promoter DNA. Recognition of the −12 nucleotide within ss DNA has also been inferred from competition binding experiments which have demonstrated the specificity of polymerase for a −12 T (Qiu and Helmann, 1999). Finally, the sensitivity of the nontemplate T at −12 to KMnO_4_, which will react with unpaired thymines and to a lesser extent cytosines, seems to depend on the particular promoter and conditions (Davis et al. 2007a; Hook-Barnard et al. 2006; Kainz and Roberts, 1992; Lim et al. 2001; Nguyen and Burgess, 1997; Sasse-Dwight and Gralla, 1989; Sasse-Dwight and Gralla, 1991; Thouvenot et al. 2004). Thus, whether an interaction between σ^70^ and position −12 in the context of ss DNA is crucial for the formation of a productive polymerase/promoter complex is not known.

### Interaction of σ^70^ region 2.3 with positions −11 through −7 of the nontemplate strand

A large body of work, detailed below, has demonstrated that polymerase recognizes the −10 element positions ^−11^ATAAT^−7^ as ss nontemplate DNA formed at the transcription bubble surrounding the start of transcription. In most of these studies, the A at −11, thought to nucleate strand opening, and the nearly invariant T at −7 have been shown to be particularly important, with lesser and varying contributions from the bases at positions −10 through −8. In many cases, these conclusions have been driven by EMSAs of polymerase and DNA performed in the absence and presence of a challenge, such as heparin. This analysis is meant to distinguish between two types of binding: 1) unstable binding of polymerase to fully duplex DNA, which should only be seen in the absence of the competitor challenge, and 2) the stable, competitor resistant complex. For most promoters, the unstable form is thought to be at an early step in initiation, like RPc or I_1_. The stable form is presumed to be at a late step, such as RPo, in which the DNA is unwound from position −11 downstream and conformational changes have resulted in the isomerization of the polymerase. In some cases, the presence of the transcription bubble has been confirmed by KMnO_4_ footprinting.

The first work to demonstrate that polymerase specifically recognizes only the nontemplate strand of the −10 element was reported by Roberts and Roberts (Roberts and Roberts, 1996). These authors investigated the ability of polymerase to form an open complex with promoter DNA containing mismatched nucleotides at the crucial −12, −11, and −7 positions on either the non-template or template strand of the −10 element. To do this analysis they performed single round transcription assays after allowing the polymerase and DNA to form heparin-resistant complexes. Mutations away from consensus on the nontemplate strand at each of these three positions were deleterious. In contrast, mutations of template strand bases at these positions were inconsequential. EMSA analysis demonstrated that polymerase will bind a ss oligomer that contains the nontemplate sequence of the −10 element (Marr and Roberts, 1997; Qiu and Helmann, 1999). In these cases, binding specificity, which was determined by competition of the complex with a mutant ss oligomer, showed that mutations away from the −10 consensus sequence impaired binding. These studies confirmed the importance of the nucleotide identity on the nontemplate strand.

Contact between polymerase and the nontemplate strand positions −11 to −7 has also been inferred in studies using fork template DNA substrates. Fork junction templates are ds DNAs with a 3′ extension on the nontemplate strand that includes part or all of the −10 element. One set of experiments has used σ^70^ labeled with (Eu^+3^)DTPA-AMCA-maleimide to assay luminescence resonance energy transfer to fork junction DNA having a 3′ nontemplate extension of −11 to −4 and labeled with the fluorophore Cy5 (Matlock and Heyduk, 2000). Binding, as assayed by the energy transfer, was measured in the presence of a non-labeled DNA containing a specific mutation. This work again indicated that the nontemplate −11 A and −7 T are particularly important for binding. In addition, substitutions of the −11 A with a series of adenine analogs argued that the N1 position of the −11 adenine is crucial for polymerase contact. This suggests that the interaction of polymerase with this base determinant could help disrupt the −11A:T bp, nucleating the strand separation that is needed for the open complex.

A series of studies from the Gralla lab has investigated the binding of polymerase to fork junction DNAs using EMSAs with or without a heparin challenge. Experiments with duplex promoter DNA from −41 to −12 with a nontemplate ss extension from −11 to −7 showed that specific base mutations within the nontemplate extension impair the formation of the stable, isomerized complex and again demonstrated the importance of the sequence of the nontemplate strand, especially at positions −11 and −7, for stable binding (Fenton and Gralla, 2001). Furthermore, even a fork junction substrate containing a single, nontemplate base 3′ extension at position −11 can form a heparin resistant complex if the −11 nucleotide is the consensus A (Guo and Gralla, 1998) or the adenine analog 2-aminopurine, which has an amino group at C2 rather than C6 (Tsujikawa et al. 2002). These results suggested that the interaction of polymerase with the nontemplate −11 A is sufficient for generating a stable polymerase/promoter complex, in which the polymerase has presumably isomerized.

Interestingly, the effect of a 2-aminopurine at −11 in duplex promoter DNA depends on the particular promoter context. In one experiment using the natural Pgal promoters, which have less than ideal promoter elements, this substitution completely inhibited strand separation (Lim et al. 2001), while in another study using an ideal promoter (a perfect −35 element, a TGn motif, and a perfect −10 element) strand separation was unaffected (Tsujikawa et al. 2002). It has been proposed that a flipping out of the −11 A nucleates strand separation, which then propagates downstream (Helmann and Chamberlin, 1988; Helmann and deHaseth, 1999; Young et al. 2004). In this model, polymerase could facilitate the flip or capture the −11 A after the flip. Thus, the ability of polymerase to engage in this process when a 2-aminopurine is present at position −11 may be influenced by the strength of the polymerase interactions with other promoter elements.

Clusters of aromatic residues can signify a ss DNA or RNA binding motif (Bochkarev et al. 1995; Burd and Dreyfuss, 1994; Helmann and Chamberlin, 1988; Shamoo et al. 1995). Structural analysis of σ^70^ region 2 (Malhotra et al. 1996) suggested that several aromatic residues lying along an α-helix face in region 2.3 might interact with ss DNA in the transcription bubble. Multiple alanine substitutions at Y425, F427, Y430, W433, and/or W434 in various combinations render polymerase significantly impaired in open, but not closed, complex formation at the lambda promoters P_R_ and P_RM_ (Cook and deHaseth, 2007; Panaghie et al. 2000). Earlier work has shown that substitutions at corresponding residues within region 2.3 of the primary σ of *B. subtilis* are defective for binding to an oligonucleotide containing the nontemplate −10 sequence (Juang and Helmann, 1994). However, it should be noted that despite the extensive analyses indicating that the ^−11^ATAAT^−7^ sequence is highly conserved, that the −11 A appears to be crucial for starting the process of strand separation, and that various aromatic residues in region 2.3 are important for ss DNA binding and polymerase function, definitive residue/base interactions have not been identified [(Fenton and Gralla, 2003; Schroeder et al. 2007) and references therein].

### Recognition of nontemplate G at position −5

Studies have indicated that nucleotides downstream of the −10 element are contacted by polymerase, and the role of σ^70^ region 1.2 in these interactions has been demonstrated (Feklistov et al. 2006; Haugen et al. 2006; Zenkin et al. 2007). At the ribosomal promoter rrnBP1, RPo is unstable, and this short half-life is a crucial factor in transcriptional regulation in response to growth conditions and nutrient availability (Paul et al. 2004). Experiments investigating this instability have revealed that the nucleotide on the nontemplate strand at position −5 can directly interact with σ^70^ region 1.2, stabilizing RPo (Haugen et al. 2006) (Fig. 1A). The identity of the nontemplate base two bases downstream of the −10 element (−5 at most promoters, but −7 at rrnBP1) is crucial in determining RPo half-life. For example, changing the nontemplate −5 nucleotide from a C to a G results in a dramatic increase in half-life at rrnBP1. Conversely, substituting the base at −5 for a C at other promoters (Pgal, λP_R_, λP_L_) significantly reduces RPo half-life. The −5G was shown to crosslink to σ^70^ region 1.2, indicating a direct contact between this region and downstream DNA. Interestingly, the crosslink between region 1.2 and the −5 base was strongest when the σ^70^ region 1.1 was missing. This result suggests that either region 1.1 blocks access to the crosslinking site, or that in the absence of region 1.1 the contact between region 1.2 and −5G is longer lived. The latter interpretation implies that the region 1.2/DNA interaction is transient and may be modulated by the movement of 1.1.

The strength of the region 1.2/−5 nucleotide interaction appears to play an important role in determining the inherent stability of RPo. The complex half-life can then be further influenced by cellular factors, such as NTP concentration, the small effector molecule ppGpp, and the protein factor DksA (Gralla, 2005; Rutherford et al. 2007). Thus, the half-life of RPo can be a major determinant of transcriptional activity and is the central conveyor of regulation at rrnBP1.

## Importance of Spacers between Elements

### The spacer between the −35 and −10 elements

In addition to the sequence determinants, RNA polymerase also has specific spacer length requirements for promoter recognition. An early analysis (Hawley and McClure, 1983) of 112 characterized bacterial and phage promoters found that 50% had a spacer of 17 bp, while 20% had a spacer of 16 bp and another 20% had a spacer of 18 bp. A more recent analysis indicated that in *E. coli* 44% of promoters have a spacer length of 17 bp, although promoters with a TGn motif are more likely to have a spacer of 18 or more bp (Mitchell et al. 2003). It is well established that promoters with a 17 bp spacer yield higher levels of transcription than otherwise identical promoters that have spacers of 16 or 18 bp (Aoyama et al. 1983; Mulligan et al. 1985; Stefano and Gralla, 1982).

The preferred spacer length between the −10 and −35 promoter elements is dictated by the distance between recognition domains within the polymerase structure. This distance is set in part, by the interaction of σ^70^ region 4 with a structure in core called the β-flap, which positions region 4 to correctly make contact with the −35 sequence while region 2 interacts with the −10 sequences (Kuznedelov et al. 2002; Murakami et al. 2002b; Vassylyev et al. 2002). The role of the sigma subunit in perceiving the spacer length was first demonstrated using free σ^70^ polypeptides and the tac promoter with spacer lengths ranging from 11 to 26 bp (Dombroski et al. 1996). σ^70^ peptides containing either region 2 or region 4 alone bound the promoter, unaffected by spacer length. However, when the polypeptide included regions 2 through 4, the length of the spacer became so important that a change of +/−1 bp rendered the promoter no more recognizable than non-promoter DNA. This work indicated that the sigma regions 4 and 2 simultaneously contact the −35 and the −10 elements. Luminescence Resonance Energy Transfer measurements have also showed that σ^70^ undergoes a conformational change upon binding to core, which alters the distance between regions 2.4 and 4.2 from 34Å apart in free σ^70^ to 50Å in holoenzyme (Callaci et al. 1998; Callaci et al. 1999). The 50Å distance between the recognition domains is conducive to interacting with −10 and −35 elements separated by the preferred 17 bp spacer.

As discussed above, polymerase is known to make contacts just upstream of the −10 element at the ^−15^TGn^−13^ motif. Although polymerase does not appear to make specific contacts with the spacer nucleotides upstream of position −15, the sequence of this spacer, in addition to its length, can affect promoter function (Chan and Busby, 1989; Chan et al. 1990; Liu et al. 2004b; Mellies et al. 1994; Repoila and Gottesman, 2003; Thouvenot et al. 2004; Warne and deHaseth, 1993). For example, changing the spacer from GC- to AT-rich sequences increases promoter activity and also affects regulation at Plac (Liu et al. 2004b) and the dsrA promoter, (Repoila and Gottesman, 2003), while individual base substitutions in the spacer affect transcription from galP1 (Chan and Busby, 1989), gapAP1 (Thouvenot et al. 2004), and the proU promoter (Mellies et al. 1994). Due to the lack of any known specific contacts, it has been assumed that the spacer sequences may affect the structure or trajectory of the promoter DNA.

### The spacer between the −10 element and position +1, the start site of transcription

The distance between the −10 element and transcriptional start site, which is constrained by the polymerase structure (Murakami et al. 2002b; Vassylyev et al. 2002), can also affect transcription levels. The preference of starting nucleotide and location relative to the −10 has been investigated in detail at the pyrC promoter (Liu and Turnbough, 1994). This study found that the preferred +1 nucleotide is A≥G>T>>C and is typically located 7>8>9 nucleotides downstream of the −7 base of the −10 element (Fig. 3). Similar results have been reported for PlacUV5 (Jeong and Kang, 1994) and the gal promoters galP1 and galP2 (Lewis and Adhya, 2004). The conclusions of these studies are consistent with those derived from a computer analysis (O’Neill, 1989) of a previous promoter set (Hawley and McClure, 1983).

## Mix and Match Elements for Binding of ds DNA Elements

### The −35/10 promoter

Promoters containing good matches to the −35 and −10 elements represent the classic group of *E. coli* promoters. Although conservation of each base pair varies somewhat within these elements, depending upon the promoters included in the data set, the relative importance of the bases is fairly consistent. The −12 T:A (79%), −11 A:T (87%), and −7 T:A (90%) bases are the most highly conserved within the −10 element (Lisser and Margalit, 1993; Mitchell et al. 2003). Within the −35 region, the −35 T:A, −34 T:A, and −33 G:C are each found at >69% of promoters (Lisser and Margalit, 1993). Interestingly, the −31 C:G bp, which is contacted directly on the template strand, is less conserved (~50%) (Lisser and Margalit, 1993). As expected, a very strong correlation between the consensus sequence and promoter activity has been demonstrated [reviewed in (Hawley and McClure, 1983)].

Several natural promoters have been studied as models for the −10/−35 class including T7A1, λ P_R_, and Plac (Fig. 3). Plac is perhaps the best characterized of all promoters and has served as a paradigm for the −10/−35 promoter class (Borukhov and Lee, 2005; Reznikoff, 1992; Silverstone et al. 1970; Stefano and Gralla, 1982). However, Plac deviates from consensus in several ways. The −35 element is TTTACA, the −10 element is TATGTT, and there is an 18 bp spacer (Fig. 3). As a result, Plac transcription is at fairly low levels until activated by CRP (c-AMP receptor protein), in response to glucose level. Several mutants of Plac, which make the −10 and/or the −35 elements more consensus, are CRP-independent (Arditti et al. 1968; Silverstone et al. 1970). Subsequent studies have demonstrated the importance of the −10, the −35, and the spacer regions for these promoters (Ackerson and Gralla, 1983; Chan and Lebowitz, 1990; Liu et al. 2004b; Makoff and Oxer, 1991; Mandecki and Reznikoff, 1982). One Plac derivative, lacUV5 (Fig. 3), has been studied extensively by genetic, biochemical, and structural methods (Stefano et al. 1980; Stefano and Gralla, 1982) [reviewed in (Borukhov and Lee, 2005; Siebenlist et al. 1980)]. The lacUV5 mutation, which changes the Plac −10 element (TATGTT) to the consensus sequence (TATAAT), increases transcription and renders lacUV5 independent of the CRP activator (Arditti et al. 1968; Silverstone et al. 1970). Comparative studies have demonstrated that, although lacUV5 is a stronger promoter, whose kinetics of RPo formation differ from Plac, the final RPo complex is quite similar to that of Plac (Meiklejohn and Gralla, 1989) (Fenton and Gralla, 2001). Footprinting of RPo at these promoters indicates that the transcription bubble is from −12/−11 to +4, and the DNA is protected from cleavage from −55 to +20 for both promoters (Sasse-Dwight and Gralla, 1989; Spassky et al. 1984; Spassky et al. 1985).

Despite their classification as −35/−10 promoters, many promoters in this class have other sequences that contribute to promoter activity. For instance, AT-rich sequences upstream of λP_R_ and T7A1 significantly increase open complex formation (Cellai et al. 2007; Davis et al. 2005; Sclavi et al. 2005). Plac (and therefore lacUV5) has a T: A bp at position −15 that influences transcription levels (Liu et al. 2004a; Munson et al. 1984). Furthermore, the T7A1 promoter has a −15 T:A and λP_R_ has a −14 G:C, which may compensate for their noncanonical bp at the highly conserved −12 position. It is well established that the effect of promoter mutations is dependent upon context and that strong polymerase-DNA contacts can compensate for weaker sites of interaction (Grana et al. 1988; Michalowski et al. 2004; Miroslavova and Busby, 2006; Moyle et al. 1991), and simply assigning a promoter by its matches to the −10 and −35 elements can fail to identify active promoters *in vivo* (Kawano et al. 2005). Thus, polymerase-promoter contacts outside of the −10 and −35 elements influence the activity of classic −10/−35 promoters and these effects should be given consideration when interpreting data.

### The TGn/-10 promoter (extended −10 promoter)

An early compilation and analysis of promoters indicated that there was some preference for the TG sequence upstream of −10 (Hawley and McClure, 1983). Genetic and biochemical data also suggested the importance of polymerase-DNA contact in this region of DNA (Berman and Landy, 1979; Busby et al. 1984; Grana et al. 1985; Ponnambalam et al. 1986; Siebenlist and Gilbert, 1980). More recently, 554 promoters of *E. coli*, whose +1 start sties had been determined and whose −10 elements had been identified, were analyzed (Burr et al. 2000; Mitchell et al. 2003). This work revealed that ^−15^TGn^−13^ is present in 20% of *E. coli* promoters. In addition, 43% of the 554 promoters have a G:C at position −14. Another 246 promoters were eliminated from the analysis because they were not well defined, leaving the possibility that the occurrence of −15T or −14G is even higher. This could be the case since promoters that deviate significantly from consensus in the −10 and −35 elements are more likely to have a TGn motif and may be less well-characterized.

Investigations into the importance of the ^−15^TGn^−13^ motif have shown that this sequence can compensate for a poor or missing −35 element. One such TGn/−10 promoter is λPre (Fig. 3), which has a very poor match to the σ^70^ −35 element, a noncanonical −10 element (^−12^AAGTAT^−7^), and requires an activator, CII, for detectable promoter function (Keilty and Rosenberg, 1987). The mutant derivative λPre* was created with a consensus −10 element, and although it still has no recognizable −35 element, λPre* is independent of CII (Keilty and Rosenberg, 1987). Both λPre and λPre* require the TGn motif, and neither promoter requires specific sequences within the −35 region (Keilty and Rosenberg, 1987). In fact, region 4.2 of σ^70^, which contacts the −35 element, can be removed entirely without eliminating transcription from λPre* (Kumar et al. 1993). In contrast, a λPre* mutant, in which the ^−15^TG^−14^ was changed to ^−15^CC^−14^, and the promoter Pcons, which has consensus −35 and −10, but no TGn, have little activity without σ^70^ region 4.2 (Kumar et al. 1993).

Another well-studied member of the TGn/−10 class is galP1 (Fig. 3). galP1 has an imperfect extended −10 sequence (TGnTATGGT) and no recognizable homology to the σ^70^ −35 element. As with λPre, the −35 region of galP1 is inessential for transcription (Ponnambalam et al. 1986), whereas the TGn is required (Busby et al. 1984). In addition, galP1 transcription is dependent on A/T rich upstream sequences or CRP for activation (Chan and Busby, 1989). Perfecting the −10 element makes galP1 independent of CRP; however, this derivative still requires TGn (Kuhnke et al. 1987; Kumar et al. 1993; Kumar et al. 1994). In contrast, creation of an ideal −35 element allows mutation of the TGn, yielding a functional and CRP independent promoter (Chan and Busby, 1989; Chan et al. 1990).

Footprinting analyses of the open complex at galP1 have indicated that the promoter region is protected from −55 to +20, similar to that observed with the −35/−10 promoters, yet there is less protection of the −35 region (Grimes et al. 1991). The protection upstream from the −35 element to −55 is dependent on the presence of the α-CTD (Burns et al. 1999). At another TGn promoter, PcysG (Fig. 3), the DNA is well protected from −55 to +20 with an additional weak protection extending upstream to −80, presumably due to interactions with the UP elements (Belyaeva et al. 1993). These results are consistent with the model that at a TGn/−10 promoter, polymerase uses other contacts to compensate for the lack of the σ^70^ region 4/−35 interaction (Busby et al. 1987). However, KMnO_4_ footprints are the same (−11 to +3) whether using galP1 or galPcon, a derivative of galP1 in which the sequences upstream of −12 have been replaced, mutating the TGn and inserting a perfect −35 element (Grimes et al. 1991). Thus, a change in upstream contacts does not appear to alter the final transcription bubble.

Unlike λPre, galP1, and PcysG, other promoters, such as those for proU (Mellies et al. 1994) (Fig. 3) and tyrT (Berman and Landy, 1979) (Fig. 3) have been characterized as extended −10 promoters, but they have reasonable matches to the −35 element, and for the tyrT promoter, this element is essential for activity (Lamond and Travers, 1983). In addition, the TGn promoter for uvrA (Fig. 3) has a relatively poor −35 element, yet this element still enhances transcription (Backendorf et al. 1983). This is also the case with the ompF promoter (Fig. 3), which has a weakly conserved −10 region (AAAGAT) and requires the TGn motif for function (Mitchell et al. 2003; Taylor et al. 1985). ompF is activated by OmpR, whose binding site overlaps the −35 region, yet transcription is also influenced by the −35 element in the absence of OmpR (Inokuchi et al. 1984; Mitchell et al. 2003; Taylor et al. 1985). Mutation of the −12 A:T to T:A makes transcription independent of the −35 region and OmpR (Dairi et al. 1985; Ozawa et al. 1987). In contrast, a ompF-tet hybrid, which has the −12 A: T to T:A change, but also has a mutated TGn, is dependent upon OmpR activation (Dairi et al. 1985). Thus, it appears that below a minimum number of polymerase contacts, i.e. a threshold, the promoter becomes activator dependent.

In summary, although extended −10 promoters have frequently been characterized as not requiring sequences upstream of position −15, this is a misleading generalization. Most of the examples discussed above require or benefit from −35 and/or UP element contacts. In addition, the majority of TGn promoters have a −35 region with at least a 3 out of 6 match to the −35 consensus sequence (Mitchell et al. 2003). As observed by Kumar et al. (Kumar et al. 1993), “there are no clear examples of “pure” extended −10 promoters lacking any −35 consensus or activator protein-binding site” (Kumar et al. 1993 p. 415). Furthermore, TGn promoters are as likely to have an imperfect −10 element as a nonconsensus −35 element (Mitchell et al. 2003). Thus, the TGn motif can compensate for poor or missing −35 contacts, but, as discussed below, it may also compensate for weak −10 interactions.

### The −35/TGn promoter

Recently, a new class of promoters has been defined, which demonstrates the role of TGn contacts in compensating for a weak −10 element. As discussed above, many promoters identified as extended −10 promoters require upstream contacts for full promoter function. It is also clear that the TGn can compensate for a −10 element with a poor match to consensus. In fact, as determined by Mitchell et al., 38% of TGn promoters have 3 or less matches within the −10 element consensus sequence, compared to 24% of non-TGn promoters (Mitchell et al. 2003).

One well-characterized −35/TGn promoter is gapAP1 (Thouvenot et al. 2004) (Fig. 3). Mutagenesis and *in vitro* transcription have demonstrated that both the −35 and TGn sequences are required for function at this promoter. These elements compensate for weak −10 interactions; when the noncanonical −10 element (^−12^AATTTT^−7^) was perfected, creating the sequence ^−15^TGnTATAAT^−7^, the −35 element was no longer necessary (Thouvenot et al. 2004). This was also the case for another −35/TGn promoter, Pminor (Hook-Barnard et al. 2006; Vuthoori et al. 2001) (Fig. 3). Both the −35 element and the TGn of Pminor are required and compensate for the poor −10 element (GAAAAC) (Hook-Barnard et al. 2006). Mutation of the −14 G:C to A:T eliminates expression, but the combination of this mutation with a −12 G:C to T:A change results in the same level of transcription as wild type Pminor. Thus the −14 G:C contact compensates for the poor −10 element and specifically for the lack of contact at −12 G:C.

Chemical probing of gapAP1 demonstrated that the polymerase-promoter interactions occur throughout the promoter region including the TGn and −35 elements. The transcription bubble was determined by KMnO_4_ footprinting to extend from −12 to +3. (The observation that the −12 is assessable for KMnO_4_ reactivity in RPo at gapAP1 is not unique to this promoter class, since this observation was also made at the −35/−10 promoter λP_R_ (Suh et al. 1993)). In addition, both the Pminor promoter and a Pminor derivative with −12 T:A yielded a transcription bubble from −11 to +3. DNase I footprinting of Pminor shows protection from −55 to +25; this is also observed with a Pminor derivative in which the −10 element has been perfected (Hook-Barnard, unpublished). Thus, the final open complex is essentially the same for all promoter types.

In addition to these well-defined −35/TGn promoters, there are other examples that have not been recognized as such. For instance, PcspA (Tanabe et al. 1992) (Fig. 3) has been described as an extended −10 promoter, because the TGn is required for transcription (Phadtare and Severinov, 2005). However, the −35 sequence (TTGCAT) is a good match to consensus and may affect promoter activity. Moreover, the −10 element (CTTAAT) is nonconsensus at the crucial −12 and −11 positions. Thus, the required TGn is as likely to compensate for the poor −10 element as the −35 element. As discussed in the previous section, many promoters, which are considered part of TGn/−10 class are dependent upon −35 sequences and have poorly functional −10 elements. Thus, assumptions should not be made about promoter/polymerase contacts with other regions simply because a promoter has or even requires a TGn sequence.

### An UP/−10 promoter (?)

Although a UP/−10 promoter has not been identified, the possibility of such a promoter has been revealed by experiments using the bacteriophage T4 protein, AsiA. AsiA binds tightly to σ^70^ region 4, dramatically changing the structure of region 4 and preventing its interaction with the −35 DNA element ((Lambert et al. 2004) reviewed in (Hinton et al. 2005)). Consequently, AsiA significantly inhibits transcription from promoters requiring a −35 element such as the −35/−10 promoter lacUV5 or the −35/TGn promoter Pminor. TGn/−10 promoters are not inhibited by AsiA (Colland et al. 1998; Pahari and Chatterji, 1997; Severinova et al. 1998). Many T4 early promoters are very strong, having portions of all four recognition elements (UP/−35/TGn/−10) and as expected, these promoters are much less susceptible to AsiA inhibition (Pene and Uzan, 2000). An analysis of what is needed to impart resistance to AsiA inhibition for one of these promoters has revealed that even if the TGn element is mutated, contact between the α-CTDs and the UP elements is still sufficient to provide significant resistance to AsiA inhibition, suggesting that in the correct context, an UP/−10 promoter is acceptable for recognition (Orsini et al. 2004). Furthermore, an extended incubation of AsiA-associated polymerase with lacUV5 or Pminor eventually results in transcriptionally competent RPo complexes at these promoters (Orsini et al. 2001; Pal et al. 2003). Given that the α-CTDs can make non-specific contacts in the −40 to −60 region, even when recognizable UP element sequences are missing, these results suggest that the extended period of incubation provides the time needed to form open complexes using these less specific contacts in the upstream region.

Another example of promoters functioning with only a −10 element and upstream contacts was demonstrated recently using a set of synthetic promoters (Miroslavova and Busby, 2006). In that study, the authors began with a promoter, which had a −10 element, but none of the other consensus sequences. As expected, expression was essentially zero. In the presence of CRP and an upstream CRP binding site, the promoter became functional even without the addition of other contact sequences, suggesting that upstream contacts plus a −10 element can be sufficient for promoter activity. Thus, although an UP element/−10 promoter has not been described, these examples indicate that such promoters may be present within the repertoire of the *E. coli* genome.

## Another Way to Consider Promoter Types: Binding/Isomerization Elements

As detailed in the first section, the earliest step in promoter recognition and binding is the formation of an unstable polymerase/promoter complex through interaction of polymerase with recognition elements present in ds DNA. Recent analyses of the earliest intermediates leading up to stable complex formation at the strong T7A1 promoter (Sclavi et al. 2005) and the λP_R_ promoter (Davis et al. 2007a) have been performed using hydroxyl radical footprinting. In these analyses, contact with the promoter initiates with the farthest upstream regions and proceeds toward the start site of transcription. Thus, the recognition of the specific ds DNA binding elements starts the process that can eventually result in the stable open complex.

Typically, a promoter has been classified depending on how well its sequence matches the consensus sequences that have been observed in σ^70^-dependent promoters (Harley and Reynolds, 1987; Hawley and McClure, 1983; Shultzaberger et al. 2007) Thus, as described above, promoters have been identified as −35/−10, TGn/−10, or more recently, −35/TGn promoters. For some promoters, such has the −35/−10 promoter lacUV5, the TGn/−10 promoter galP1, and the −35/TGn promoter Pminor, these classifications seem reasonable (Fig. 3). Matches to the identified consensus sequences are clear, and biochemical analyses have indicated that the designations actually reflect the regions that are important for the function of the promoter. However, for most promoters this seems like an arbitrary system of classification for several reasons. First, many promoter sequences have a mixture of various potential ds binding elements that could contribute to binding (Mitchell et al. 2003) (Fig. 3). TGn/−10 promoters often have a recognizable −35 element, and this −35 element can contribute to transcriptional activity (Miroslavova and Busby, 2006; Mitchell et al. 2003). In addition, UP elements can improve the activity of a TGn/−10 promoter in the absence of a good −35 element (Miroslavova and Busby, 2006), and improvement of the −10 element of the −35/TGn promoter Pminor increases its activity (Hook-Barnard et al. 2006). Many promoters that are placed in the −35/−10 class have either a −15 T:A or −14 G:C and it has been shown that having one match to the TGn element can improve activity in some cases (Mitchell et al. 2003). The second reason that the present promoter classification is somewhat misleading is because the −10 element, which is used as one of the classifications, is fundamentally different from the other elements. Accumulated evidence suggests that the −12 bp is primarily a ds recognition element for the −10 region and the −11 to −7 base pairs contribute little to specific duplex binding. Recognition of base determinants in the sequences downstream of position −12 occurs during and after polymerase isomerization. Thus, the −10 element is not a single recognition element. Rather it should be considered as two elements: a −10 binding element (primarily position −12) and a −10 melting element (positions −11 to −7). Finally, regardless of the promoter classification, the final transcription bubble in RPo appears to be the same. KMnO_4_ analyses have indicated that once the open complex is formed, the unpaired thymines always extend from −12 or −11 to around +3 (Davis et al. 2007a; Grimes et al. 1991; Hook-Barnard et al. 2006; Kainz and Roberts, 1992; Lim et al. 2001; Nguyen and Burgess, 1997; Sasse-Dwight and Gralla, 1989; Sasse-Dwight and Gralla, 1991; Thouvenot et al. 2004). In fact, even a minimal promoter, a short DNA duplex from −18 to −5 that only contains a consensus −10 element (without a TGn motif), will eventually produce a transcription bubble after a long incubation with polymerase (Niedziela-Majka and Heyduk, 2005). Although this is an inefficient process, it illustrates the point that the initial binding contacts do not qualitatively influence the final stable complex. Instead, all the contacts needed for isomerization are contained in the −10 element. Furthermore, other work has shown that a minimal polymerase, containing only σ^70^ regions 2 and 3 and just a portion of core, is capable of forming an open complex with an extended −10 promoter (Young et al. 2004). This finding is also consistent with the idea that minimal polymerase/DNA contacts are acceptable for isomerization. Thus, the strength and number of binding contacts appear to affect the kinetics of recognition and isomerization without substantively affecting the salient features of the final open complex.

Taken together, the data argue that there are 3 promoter locations that can be used for ds binding: UP elements, the −35 element and a −15 element, ^−17^tgTGnT^−12^. (The −15 element is a combination of the TGn element with the −12T.) Certainly a minimal number of contacts within these ds binding elements are needed for sufficient recognition and binding, but various combinations are absolutely permitted. Thus, the ds binding elements represent a set of mix and match, or as designated by the Busby lab: modular (Miroslavova and Busby, 2006) elements, which appear to work interchangeably for the early steps in transcription initiation. The subsequent steps then rely on interactions with ss elements, the ^−11^ATAAT^−7^ and the −5 G, which interact with σ^70^ regions 2.3 and 1.2 respectively.

Interestingly, a fully consensus promoter is undesirable in several ways. First, a number of studies have indicated that having too may contacts actually reduces transcriptional activity for a promoter, presumably because they impede the transition from the open complex to promoter clearance and elongation (Grana et al. 1988; Miroslavova and Busby, 2006). Thus, promoters have evolved to contain the optimal number of contacts such that the promoter not only can be recognized, but will also be released as transcription proceeds. Second, a less than canonical sequence provides the opportunity for regulation. Promoters that are appropriately responsive to cellular signals provide a selective advantage, and are favored over promoters that are intrinsically high functioning, but unregulated. As we learn more about the steps leading to transcription, we see that a promoter not only supplies information via its consensus sequences, but that the nature of noncanonical sequences may provide insight into the form of regulation required to trigger activity. For instance, promoters with relatively few matches to ds binding elements may remain latent until an activator supplies the additional contacts that strengthen initial binding. In contrast, promoters with mismatches in the −11 to −7 region may require activators, or conditions (temperature, salt, supercoiling) that facilitate DNA melting, or polymerase isomerization. Furthermore, a nonpreferred nucleotide at −7 or −5 may indicate that the RPo is unstable and therefore responsive to modulating factors such as DksA, ppGpp, or NTP concentration (Rutherford et al. 2007). Thus, the ability of a promoter to respond to cellular conditions is absolutely dependent upon its flaws, and the mix and match nature of promoter elements allows a wide variety of ways to arrive at the desired outcome: effective transcription. Consequently, perfect promoters are not biologically relevant.

## Abbreviations

ss: single-stranded
ds: double-stranded
EMSA: electrophoretic mobility shift assay
bp: base pair
n: any nucleotide
r: purine
y: pyrimidine
w: A or T
NTP: ribonucleoside triphosphate
